# Redetermination of AgPO_3_

**DOI:** 10.1107/S1600536811003977

**Published:** 2011-02-09

**Authors:** Katherina V. Terebilenko, Igor V. Zatovsky, Ivan V. Ogorodnyk, Vyacheslav N. Baumer, Nikolay S. Slobodyanik

**Affiliations:** a Department of Inorganic Chemistry, Taras Shevchenko National University, 64 Volodymyrska Street, 01601 Kyiv, Ukraine; b STC ‘Institute for Single Crystals’, NAS of Ukraine, 60 Lenin Avenue, 61001 Kharkiv, Ukraine

## Abstract

Single crystals of silver(I) polyphosphate(V), AgPO_3_, were prepared *via* a phospho­ric acid melt method using a solution of Ag_3_PO_4_ in H_3_PO_4_. In comparison with the previous study based on single-crystal Weissenberg photographs [Jost (1961 ▶). *Acta Cryst*. **14**, 779–784], the results were mainly confirmed, but with much higher precision and with all displacement parameters refined anisotropically. The structure is built up from two types of distorted edge- and corner-sharing [AgO_5_] polyhedra, giving rise to multidirectional ribbons, and from two types of PO_4_ tetra­hedra linked into meandering chains (PO_3_)_*n*_ spreading parallel to the *b* axis with a repeat unit of four tetra­hedra. The calculated bond-valence sum value of one of the two Ag^I^ ions indicates a significant strain of the structure.

## Related literature

For a previous crystallographic study of AgPO_3_, see: Jost (1961 ▶). For the isotypic *A*-form of the Kurrol salt NaPO_3_, see: McAdam *et al.* (1968 ▶). Properties of glassy silver phosphates have been reported by Portier *et al.* (1990 ▶) and Novita *et al.* (2009 ▶). For long-chain polyphosphates Ag*M* ^III^(PO_3_)_4_ (*M* ^III^ = La, Gd, Eu), see: El Masloumi *et al.* (2005 ▶); Naıli *et al.* (2006 ▶); Ayadi *et al.* (2009 ▶). For Ag*M* ^II^ (PO_3_)_3_ (*M* ^II^ = Mg, Zn, Ba), see: Belharouak *et al.* (1999 ▶); for Ag*M* ^I^(PO_3_)_2_ (*M* ^I^ = K, Rb, Cs, Tl), see: Averbuch-Pouchot (1993 ▶). For background to the bond-valence method, see: Brown & Altermatt (1985 ▶).

## Experimental

### 

#### Crystal data

AgPO_3_
*M* *_r_* = 186.84
Monoclinic, 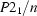

*a* = 11.9335 (3) Å
*b* = 6.0667 (1) Å
*c* = 7.3278 (2) Å
β = 93.491 (2)°
*V* = 529.53 (2) Å^3^
*Z* = 8
Mo *K*α radiation
μ = 7.96 mm^−1^
*T* = 293 K
0.10 × 0.08 × 0.04 mm

#### Data collection

Oxford Diffraction Xcalibur-3 CCD diffractometer
Absorption correction: multi-scan (Blessing, 1995 ▶) *T*_min_ = 0.465, *T*_max_ = 0.733
22720 measured reflections
2333 independent reflections
2208 reflections with *I* > 2σ(*I*)
*R*_int_ = 0.042

#### Refinement

*R*[*F* ^2^ > 2σ(*F* ^2^)] = 0.024
*wR*(*F* ^2^) = 0.058
*S* = 1.08
2333 reflections
92 parameters
Δρ_max_ = 1.43 e Å^−3^
Δρ_min_ = −1.86 e Å^−3^

### 

Data collection: *CrysAlis CCD* (Oxford Diffraction, 2006 ▶); cell refinement: *CrysAlis CCD*; data reduction: *CrysAlis RED* (Oxford Diffraction, 2006 ▶); program(s) used to solve structure: *SHELXS97* (Sheldrick, 2008 ▶); program(s) used to refine structure: *SHELXL97* (Sheldrick, 2008 ▶); molecular graphics: *DIAMOND* (Brandenburg, 1999 ▶); software used to prepare material for publication: *WinGX* (Farrugia, 1999 ▶) and *enCIFer* (Allen *et al.*, 2004 ▶).

## Supplementary Material

Crystal structure: contains datablocks global, I. DOI: 10.1107/S1600536811003977/wm2454sup1.cif

Structure factors: contains datablocks I. DOI: 10.1107/S1600536811003977/wm2454Isup2.hkl

Additional supplementary materials:  crystallographic information; 3D view; checkCIF report

## Figures and Tables

**Table:** 

Ag1—O3^i^	2.441 (2)
Ag1—O1	2.460 (2)
Ag1—O6^ii^	2.491 (2)
Ag1—O1^iii^	2.511 (2)
Ag1—O6	2.540 (2)
Ag2—O5^iv^	2.3708 (19)
Ag2—O5^v^	2.3756 (19)
Ag2—O3	2.3968 (19)
Ag2—O6^ii^	2.487 (2)
Ag2—O3^iv^	2.750 (2)
P1—O1	1.490 (2)
P1—O3	1.4952 (19)
P1—O4^vi^	1.5889 (17)
P1—O2	1.6033 (17)
P2—O5	1.479 (2)
P2—O6	1.4924 (19)
P2—O4	1.5909 (17)
P2—O2	1.6074 (18)

**Table:** 

P1—O2—P2	124.88 (11)
P1^vii^—O4—P2	135.91 (11)

Symmetry codes: (i) 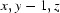; (ii) 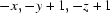; (iii) 
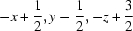
; (iv) 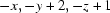; (v) 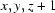; (vi) 
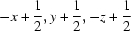
; (vii) 
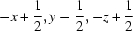
.

**Fig. 1.**
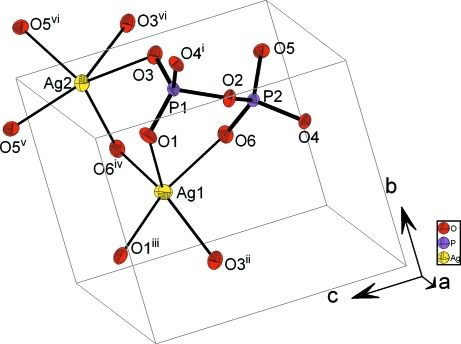
The connectivity of the Ag and P atoms in the structure of AgPO3 with displacement ellipsoids disolayed at the 50% probability level. [Symmetry codes: (i) 0.5 - x, 1/2 + y, 0.5 - z; (ii) -x, -1 + y, z; (iii) 0.5 - x,1/2 + y, 1.5 - z; (iv) -x, 1 - y, 1 - z; (v) x, y, 1 + z; (vi) -x, 2 - y, 1 - z;].

**Fig. 2.**
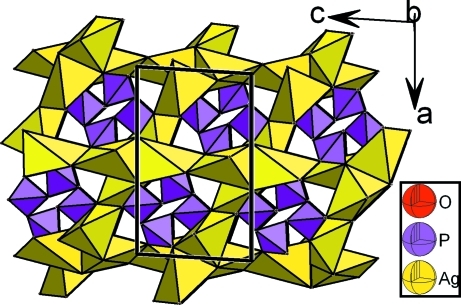
A view of the crystal structure of AgPO3 down the b-axis, emphasizing the tunnels with eight-sided windows where the helical polyphosphate chains reside.

## supplementary crystallographic information

### Comment

Glassy silver polyphosphates as a part of complex oxide-chalcogenide systems have various applications in the field of solid electrolytes (Portier *et al.*, 1990; Novita *et al.*, 2009). Much attention has therefore been paid to phase equilibrium studies within AgPO_3_–*M*(PO_3_)_n_ systems, where *M* is a rare earth (Ayadi *et al.*, 2009; Naili *et al.*, 2006, El Masloumi *et al.*, 2005), a divalent (Belharouak *et al.*, 1999) or a monovalent metal (Averbuch-Pouchot, 1993).

The title compound is isotypic with the *A*-form of the Kurrol salt NaPO_3_ (McAdam *et al.*, 1968). AgPO_3_ has been previously structurally studied based on single crystal Weissenberg photographs (Jost, 1961). The current study mainly confirms the previous results, but with significantly higher precision and with anisotropic displacement parameters refined for all atoms.

The structure of AgPO_3_ features two types of penta-coordinated Ag^I^ ions: Ag1 is located in a distorted trigonal bipyramid and Ag2 in a irregular tetragonal pyramid of oxygen atoms (Fig. 1). Calculated values of bond valence sums (BVS; Brown & Altermatt, 1985) were found to be quite different: 0.96 valence units (v.u.) for Ag2 and 0.87 v.u. for Ag1. The observed deviation of the BVS of the latter atom from its chemical valence (expected 1) is due to a high polyhedral distortion caused by the presence of rigid (PO_3_)*_n_* chains. In this case the BVS values may be seen as a degree of silver "underbonding" and have a concomitant effect on physical properties of the title compound or glassy materials containing silver polyphosphate. The next-nearest coordination spheres of Ag1 include six adjacent silver atoms, resulting in edge-sharing Ag1 and Ag2 polyhedra and other four polyhedra connected through corners. As a result of this linkage, two polyhedral ribbons appear. One spreads parallel to the *a*-axis through interconnected [Ag1O_5_] polyhedra by sharing a common edge and vertex alternatively, and another spreads parallel to the *b*-axis and consist of corner-sharing [Ag1O_5_] polyhedra. Ag2 is remotely surrounded by one Ag1 and two Ag2, resulting in a ribbon of edge-sharing  [Ag2O_5_] polyhedra that run parallel to the *c*-axis. Intersecting these ribbons leads to a three-dimensional network penetrated with tunnels having eight-sided windows. Helical chains (PO_3_)*_n_* with a repeating unit of four phosphate tetrahedra are located within the tunnels and spiral along the 2_1_ axes parallel to the *b*-axis. Due to the centrosymmetric nature of the structure, adjacent chains are left- and right-helices. As is characteristic for *catena*-polyphosphates (Averbuch-Pouchot, 1993), each of the two PO_4_ tetrahedra displays two types of P—O bond lengths: P—O terminal ranging from 1.479 (2) to 1.4952 (19) Å and P—O bridging from 1.5889 (17) to 1.6074 (18) Å. The corresponding BVS are 4.92 v.u. and 4.95 v.u. for P1 and P2, respectively. The BVS per doubled formula (corresponds to the crystallographically independent atoms in a cell) of positively charged atoms equals to 11.72 v.u., while the chemical charge of the remaining O atoms is equal to -12 valences. This difference is an additional indication of the strain in the structure caused by the presence of rigid phosphate chains.

### Experimental

AgPO_3_ was prepared by crystallizing a solution of Ag_3_PO_4_ in H_3_PO_4_ (84 %_wt_) at a molar ratio Ag/P = 0.01. The thermal treatment included heating the mixture in a graphite crucible at 473 K for 6 h and then cooling to room temperature. After leaching with water, the product consisted of colorless prismatic crystals.

### Refinement

The highest peak and the deepest hole in the final difference map are located at 0.56 Å from Ag1 (1.432 e/Å^3^) and 0.56 Å from Ag1 (-1.858 e/Å^3^), respectively

### Crystal data

**Table:**

AgPO_3_	*F*(000) = 688
*M**_r_* = 186.84	*D*_x_ = 4.687 Mg m^−^^3^
Monoclinic, *P*2_1_/*n*	Mo *K*α radiation, λ = 0.71073 Å
Hall symbol: -P 2yn	Cell parameters from 22720 reflections
*a* = 11.9335 (3) Å	θ = 3.2–35.0°
*b* = 6.0667 (1) Å	µ = 7.96 mm^−^^1^
*c* = 7.3278 (2) Å	*T* = 293 K
β = 93.491 (2)°	Prism, colorless
*V* = 529.53 (2) Å^3^	0.10 × 0.08 × 0.04 mm
*Z* = 8	

### Data collection

**Table:**

Oxford Diffraction Xcalibur-3 CCD diffractometer	2333 independent reflections
Radiation source: fine-focus sealed tube	2208 reflections with *I* > 2σ(*I*)
graphite	*R*_int_ = 0.042
φ and ω scans	θ_max_ = 35°, θ_min_ = 3.2°
Absorption correction: multi-scan (Blessing, 1995)	*h* = −19→19
*T*_min_ = 0.465, *T*_max_ = 0.733	*k* = −9→9
22720 measured reflections	*l* = −11→11

### Refinement

**Table:**

Refinement on *F*^2^	Primary atom site location: structure-invariant direct methods
Least-squares matrix: full	Secondary atom site location: difference Fourier map
*R*[*F*^2^ > 2σ(*F*^2^)] = 0.024	*w* = 1/[σ^2^(*F*_o_^2^) + (0.0212*P*)^2^ + 1.7313*P*] where *P* = (*F*_o_^2^ + 2*F*_c_^2^)/3
*wR*(*F*^2^) = 0.058	(Δ/σ)_max_ = 0.001
*S* = 1.08	Δρ_max_ = 1.43 e Å^−^^3^
2333 reflections	Δρ_min_ = −1.86 e Å^−^^3^
92 parameters	Extinction correction: *SHELXL97* (Sheldrick, 2008)
0 restraints	Extinction coefficient: 0.0034 (3)

### Special details

**Table:**

Geometry. All e.s.d.'s (except the e.s.d. in the dihedral angle between two l.s. planes) are estimated using the full covariance matrix. The cell e.s.d.'s are taken into account individually in the estimation of e.s.d.'s in distances, angles and torsion angles; correlations between e.s.d.'s in cell parameters are only used when they are defined by crystal symmetry. An approximate (isotropic) treatment of cell e.s.d.'s is used for estimating e.s.d.'s involving l.s. planes.
Refinement. Refinement of *F*^2^ against ALL reflections. The weighted *R*-factor *wR* and goodness of fit *S* are based on *F*^2^, conventional *R*-factors *R* are based on *F*, with *F* set to zero for negative *F*^2^. The threshold expression of *F*^2^ > σ(*F*^2^) is used only for calculating *R*-factors(gt) *etc*. and is not relevant to the choice of reflections for refinement. *R*-factors based on *F*^2^ are statistically about twice as large as those based on *F*, and *R*- factors based on ALL data will be even larger.

### Fractional atomic coordinates and isotropic or equivalent isotropic displacement parameters (Å^2^)

**Table:**

	*x*	*y*	*z*	*U*_iso_*/*U*_eq_	
Ag1	0.127313 (18)	0.33468 (3)	0.61230 (3)	0.02461 (6)	
Ag2	0.03023 (2)	0.89757 (4)	0.77035 (3)	0.02748 (6)	
P1	0.22766 (5)	0.81768 (10)	0.48624 (7)	0.01372 (10)	
P2	0.11152 (5)	0.61417 (10)	0.18002 (8)	0.01331 (10)	
O1	0.25346 (18)	0.6554 (3)	0.6355 (3)	0.0241 (4)	
O2	0.22388 (14)	0.6980 (3)	0.2909 (2)	0.0179 (3)	
O3	0.12509 (15)	0.9578 (3)	0.4956 (3)	0.0218 (3)	
O4	0.16402 (14)	0.4679 (3)	0.0265 (2)	0.0169 (3)	
O5	0.05294 (17)	0.7997 (3)	0.0843 (3)	0.0234 (3)	
O6	0.04724 (16)	0.4744 (3)	0.3047 (3)	0.0220 (3)	

### Atomic displacement parameters (Å^2^)

**Table:**

	*U*^11^	*U*^22^	*U*^33^	*U*^12^	*U*^13^	*U*^23^
Ag1	0.02707 (10)	0.02043 (10)	0.02611 (10)	0.00056 (7)	−0.00023 (7)	0.00252 (7)
Ag2	0.03883 (12)	0.02340 (11)	0.02121 (9)	0.00423 (8)	0.01011 (8)	0.00068 (7)
P1	0.0138 (2)	0.0153 (2)	0.0121 (2)	−0.00206 (17)	0.00169 (16)	0.00000 (17)
P2	0.0124 (2)	0.0141 (2)	0.0134 (2)	0.00133 (17)	0.00096 (16)	−0.00069 (17)
O1	0.0312 (9)	0.0223 (9)	0.0183 (7)	−0.0081 (7)	−0.0021 (7)	0.0064 (6)
O2	0.0155 (6)	0.0225 (8)	0.0157 (6)	−0.0010 (6)	0.0008 (5)	−0.0059 (6)
O3	0.0169 (7)	0.0268 (9)	0.0220 (8)	0.0031 (6)	0.0041 (6)	−0.0047 (7)
O4	0.0161 (6)	0.0200 (8)	0.0145 (6)	0.0058 (6)	0.0000 (5)	−0.0042 (5)
O5	0.0278 (9)	0.0208 (8)	0.0211 (8)	0.0108 (7)	−0.0020 (6)	0.0000 (6)
O6	0.0218 (8)	0.0243 (9)	0.0204 (7)	−0.0050 (7)	0.0061 (6)	0.0000 (7)

### Geometric parameters (Å, °)

**Table:**

Ag1—O3^i^	2.441 (2)	P1—O4^vii^	1.5889 (17)
Ag1—O1	2.460 (2)	P1—O2	1.6033 (17)
Ag1—O6^ii^	2.491 (2)	P2—O5	1.479 (2)
Ag1—O1^iii^	2.511 (2)	P2—O6	1.4924 (19)
Ag1—O6	2.540 (2)	P2—O4	1.5909 (17)
Ag1—Ag2^i^	3.1431 (3)	P2—O2	1.6074 (18)
Ag2—O5^iv^	2.3708 (19)	O1—Ag1^viii^	2.511 (2)
Ag2—O5^v^	2.3756 (19)	O3—Ag1^vi^	2.441 (2)
Ag2—O3	2.3968 (19)	O4—P1^ix^	1.5889 (17)
Ag2—O6^ii^	2.487 (2)	O5—Ag2^iv^	2.3708 (19)
Ag2—O3^iv^	2.750 (2)	O5—Ag2^x^	2.3756 (19)
Ag2—Ag1^vi^	3.1431 (3)	O6—Ag2^ii^	2.487 (2)
P1—O1	1.490 (2)	O6—Ag1^ii^	2.491 (2)
P1—O3	1.4952 (19)		
			
O3^i^—Ag1—O1	139.36 (7)	O3—P1—O4^vii^	110.36 (11)
O3^i^—Ag1—O6^ii^	121.99 (6)	O1—P1—O2	110.46 (11)
O1—Ag1—O6^ii^	97.63 (7)	O3—P1—O2	108.66 (10)
O3^i^—Ag1—O1^iii^	81.11 (6)	O4^vii^—P1—O2	100.72 (9)
O1—Ag1—O1^iii^	88.56 (4)	O5—P2—O6	118.48 (12)
O6^ii^—Ag1—O1^iii^	117.80 (6)	O5—P2—O4	106.52 (10)
O3^i^—Ag1—O6	90.36 (7)	O6—P2—O4	110.79 (11)
O1—Ag1—O6	89.59 (6)	O5—P2—O2	110.76 (12)
O6^ii^—Ag1—O6	77.68 (6)	O6—P2—O2	108.33 (10)
O1^iii^—Ag1—O6	164.51 (6)	O4—P2—O2	100.46 (9)
O3^i^—Ag1—Ag2^i^	48.87 (5)	P1—O1—Ag1	111.96 (11)
O1—Ag1—Ag2^i^	151.61 (4)	P1—O1—Ag1^viii^	109.67 (10)
O6^ii^—Ag1—Ag2^i^	88.27 (5)	Ag1—O1—Ag1^viii^	135.28 (8)
O1^iii^—Ag1—Ag2^i^	64.42 (5)	P1—O2—P2	124.88 (11)
O6—Ag1—Ag2^i^	118.78 (4)	P1—O3—Ag2	112.45 (11)
O5^iv^—Ag2—O5^v^	77.57 (7)	P1—O3—Ag1^vi^	123.86 (11)
O5^iv^—Ag2—O3	119.43 (7)	Ag2—O3—Ag1^vi^	81.04 (6)
O5^v^—Ag2—O3	145.04 (7)	P1^ix^—O4—P2	135.91 (11)
O5^iv^—Ag2—O6^ii^	129.94 (7)	P2—O5—Ag2^iv^	125.12 (11)
O5^v^—Ag2—O6^ii^	90.36 (7)	P2—O5—Ag2^x^	132.23 (11)
O3—Ag2—O6^ii^	98.10 (6)	Ag2^iv^—O5—Ag2^x^	102.43 (7)
O5^iv^—Ag2—Ag1^vi^	71.68 (5)	P2—O6—Ag2^ii^	125.33 (11)
O5^v^—Ag2—Ag1^vi^	123.01 (5)	P2—O6—Ag1^ii^	110.73 (11)
O3—Ag2—Ag1^vi^	50.09 (5)	Ag2^ii^—O6—Ag1^ii^	99.83 (6)
O6^ii^—Ag2—Ag1^vi^	145.52 (4)	P2—O6—Ag1	123.59 (11)
O1—P1—O3	118.36 (12)	Ag2^ii^—O6—Ag1	90.46 (7)
O1—P1—O4^vii^	106.84 (10)	Ag1^ii^—O6—Ag1	102.32 (6)

Symmetry codes: (i) *x*, *y*−1, *z*; (ii) −*x*, −*y*+1, −*z*+1; (iii) −*x*+1/2, *y*−1/2, −*z*+3/2; (iv) −*x*, −*y*+2, −*z*+1; (v) *x*, *y*, *z*+1; (vi) *x*, *y*+1, *z*; (vii) −*x*+1/2, *y*+1/2, −*z*+1/2; (viii) −*x*+1/2, *y*+1/2, −*z*+3/2; (ix) −*x*+1/2, *y*−1/2, −*z*+1/2; (x) *x*, *y*, *z*−1.
